# Computation of Entropy Measures for Metal-Organic Frameworks

**DOI:** 10.3390/molecules28124726

**Published:** 2023-06-13

**Authors:** Muhammad Imran, Abdul Rauf Khan, Mohamad Nazri Husin, Fairouz Tchier, Muhammad Usman Ghani, Shahid Hussain

**Affiliations:** 1 Department of Mathematical Sciences, United Arab Emirates University, Al Ain P. O. Box 15551, United Arab Emirates; 2 Department of Mathematics, Faculty of Science, Ghazi University, Dera Ghazi Khan 32200, Pakistan; 3 Special Interest Group on Modelling, Data Analytics (SIGMDA) Faculty of Ocean Engineering Technology, Informatics Universiti Malaysia Terengganu, Kuala Nerus 21030, Terengganu, Malaysia; 4 Mathematics Department, College of Science, King Saud University, P.O. Box 22452, Riyadh 11495, Saudi Arabia; ftchier@ksu.edu.sa; 5 Institute of Mathematics, Khawaja Fareed University of Engineering & Information Technology, Abu Dhabi Road, Rahim Yar Khan 64200, Pakistan; usmanghani85a@gmail.com; 6 Energy Engineering Division, Department of Engineering Science and Mathematics, Lulea University of Technology, 97187 Lulea, Sweden; shahid.hussain@associated.ltu.se

**Keywords:** FeTPyP, topological indices, CoBHT (CO), *K*-Banhatti entropies, atom-bond sum connectivity entropy, metal-organic framework, calculations, molecular graph, redefined Zagreb entropies

## Abstract

Entropy is a thermodynamic function used in chemistry to determine the disorder and irregularities of molecules in a specific system or process. It does this by calculating the possible configurations for each molecule. It is applicable to numerous issues in biology, inorganic and organic chemistry, and other relevant fields. Metal–organic frameworks (MOFs) are a family of molecules that have piqued the curiosity of scientists in recent years. They are extensively researched due to their prospective applications and the increasing amount of information about them. Scientists are constantly discovering novel MOFs, which results in an increasing number of representations every year. Furthermore, new applications for MOFs continue to arise, illustrating the materials’ adaptability. This article investigates the characterisation of the metal–organic framework of iron(III) tetra-p-tolyl porphyrin (FeTPyP) and CoBHT (CO) lattice. By constructing these structures with degree-based indices such as the K-Banhatti, redefined Zagreb, and the atom-bond sum connectivity indices, we also employ the information function to compute entropies.

## 1. Introduction

Molecular organic frameworks are compounds composed of a central metal ion or atom surrounded by one or more organic ligands [1]. These ligands are typically organic molecules with a functional group that can bind to the metal center through covalent or coordinate bonds. The resulting structure is a complex in which the metal ion or atom is coordinated to the ligands and surrounded by a coordination sphere [2]. Molecular organic frameworks have many applications [3], including catalysis [4], sensing [5], and molecular recognition [6]. For example, some metalloenzyme active sites are molecular organic frameworks, and the coordination of the metal ion or atom to the ligands plays a critical role in the enzyme’s function. In addition to their practical applications, molecular organic frameworks are also studied for their fundamental chemical properties and as models for more complex systems. The structures of molecular organic frameworks can be determined using techniques such as X-ray crystallography, and their reactivity and stability can be studied through various chemical and spectroscopic methods [7]. Molecular organic frameworks have a wide range of applications due to their unique properties, such as catalytic activity, electronic conductivity [8], and magnetic behavior [9]. Some of the applications of molecular organic frameworks are catalysis. Molecular organic frameworks are widely used as catalysts in various chemical reactions [10]. The ligands surrounding the central metal atom or ion can modify its electronic properties and facilitate the reaction by lowering the activation energy required. For example, the ruthenium-based Grubbs’ catalyst is a molecular organic framework widely used in olefin metathesis reactions [11]. Molecular organic frameworks can be designed to detect specific analytes [12], such as metal ions or small molecules, by incorporating ligands with selective binding properties. The complex undergoes a change in its optical, electronic, or magnetic properties upon binding to the analyte, which can be detected and quantified [13]. Molecular organic frameworks can be designed to recognize and bind specific target molecules, such as biomolecules, by incorporating ligands with complementary binding sites. This can be useful for developing biosensors [14] or drug discovery [15]. Molecular organic frameworks with conductive ligands can be used in organic light emitting diodes [16] and organic photovoltaics [17] due to their ability to transport charge and emit light. Overall, the unique properties of molecular organic frameworks make them versatile materials with applications in various fields, including chemistry, biology, and materials science [18]. The optical properties of the metallic nanoparticles are of interest to scientists and researchers. The nanoparticles’ heat disintegrates malignant tissue while sparing healthy cells. Niobium nanoparticles are ideal for optothermal cancer treatment because of their fast ligand binding [19]. Scientists have been fascinated by chemical graph theory, an emerging discipline of applied chemistry, for the past 20 years [20,21,22,23]. In this field of study, substantial discoveries have been made by scientists, including [24,25,26,27,28,29,30]. Using combinatorial techniques such as vertex and edge partitions, we look into the interaction between atoms and bonds. In order to provide instructions for treating malignancies or tumours, topological indices are crucial. These indices can be discovered numerically or experimentally. Although expensive, experimental data are valuable; consequently, computer analysis provides a time- and cost-effective option.

A topological index is created by converting a chemical structure into a number [31]. The topological index is a graph invariant that describes the topology of the graph and is true even during graph automorphism. A topological index is a number that can only be expressed in terms of the graph. In chemical graph theory, the eccentricity-based topological indices are essential [32]. By investigating the connection between a specific hydrocarbon compound’s molecular structure and its physical and chemical properties in 1947, a chemist named Wiener developed a topological index for the first time [33]. The second Zagreb index was redefined in 2010, and Damir et al. determined that it was identical to the inverse sum indeg index [34].

We applied valency-based entropies in this article, where $v_{1}$ and $v_{2}$ denote the valency of atoms, $b_{1}$ and $b_{2}$, within the molecule. With the use of several Banhatti indices and the valency of atom bonds, Kulli began computing valency-based topological indices in 2016 [35,36,37], all of which are defined as follows:

The *K*-Banhatti polynomial and index are:

(1) $$\;B_{1}\left(T,s\right)=\sum_{{\dot{g}}_{1}∼{\dot{g}}_{2}}s^{\left(w_{{\dot{g}}_{1}}+w_{{\dot{g}}_{2}}\right)}\;\;\;\;\;\;B_{1}\left(T\right)=\sum_{{\dot{g}}_{1}∼{\dot{g}}_{2}}\left(w_{{\dot{g}}_{1}}+w_{{\dot{g}}_{2}}\right)$$

The second *K*-Banhatti polynomial and index are:

(2) $$\;B_{2}\left(T,s\right)=\sum_{{\dot{h}}_{1}∼{\dot{h}}_{2}}s^{\left(w_{1}\times w_{{\dot{g}}_{2}}\right)}\;\;\;\;\;\;B_{2}\left(T\right)=\sum_{{\dot{h}}_{1}∼{\dot{h}}_{2}}\left(w_{1}\times w_{{\dot{g}}_{2}}\right)$$

The first hyper *K*-Banhatti polynomial and index are:

(3) $$\;HB_{1}\left(T,s\right)=\sum_{{\dot{g}}_{1}∼{\dot{g}}_{2}}s^{{\left(w_{{\dot{g}}_{1}}+w_{{\dot{g}}_{2}}\right)}^{2}}\;\;\;\;\;\;HB_{1}\left(T\right)=\sum_{{\dot{g}}_{1}∼{\dot{g}}_{2}}{\left(w_{{\dot{g}}_{1}}+w_{{\dot{g}}_{2}}\right)}^{2}$$

(4) $$\;HB_{2}\left(T,s\right)=\sum_{{\dot{g}}_{1}∼{\dot{g}}_{2}}s^{{\left(w_{{\dot{g}}_{1}}\times w_{{\dot{g}}_{2}}\right)}^{2}}\;\;\;\;\;\;HB_{2}\left(T\right)=\sum_{{\dot{g}}_{1}∼{\dot{g}}_{2}}{\left(w_{{\dot{g}}_{1}}\times w_{{\dot{g}}_{2}}\right)}^{2}$$

The concept of Redefined Zagreb indices was initiated by Ranjini in [38], and Shanmukha in [39] and defined as

(5) $$\;\;ReZG_{1}\left(T,s\right)=\sum_{{\dot{g}}_{1}∼{\dot{g}}_{2}}s^{\frac{w_{{\dot{g}}_{1}}+w_{{\dot{g}}_{2}}}{w_{{\dot{g}}_{1}}\times w_{{\dot{g}}_{2}}}}\;\;\;\;\;\;ReZG_{1}=\sum_{{\dot{g}}_{1}∼{\dot{g}}_{2}}\frac{w_{{\dot{g}}_{1}}+w_{{\dot{g}}_{2}}}{w_{{\dot{g}}_{1}}\times w_{{\dot{g}}_{2}}}.$$

(6) $$\;\;ReZG_{2}\left(T,s\right)=\sum_{{\dot{g}}_{1}∼{\dot{g}}_{2}}s^{\frac{w_{{\dot{g}}_{1}}\times w_{{\dot{g}}_{2}}}{w_{{\dot{g}}_{1}}+w_{{\dot{g}}_{2}}}}\;\;\;\;\;\;ReZG_{2}=\sum_{{\dot{g}}_{1}∼{\dot{g}}_{2}}\frac{w_{{\dot{g}}_{1}}\times w_{{\dot{g}}_{2}}}{w_{{\dot{g}}_{1}}+w_{{\dot{g}}_{2}}}.$$

The third redefined Zagreb index was defined as

(7) $$\;ReZG_{3}\left(T,s\right)=\sum_{{\dot{g}}_{1}∼{\dot{g}}_{2}}s^{\left(w_{{\dot{g}}_{1}}\times w_{{\dot{g}}_{2}}\right)\left(w_{{\dot{g}}_{1}}+w_{{\dot{g}}_{2}}\right)}\;\;ReZG_{3}=\sum_{{\dot{g}}_{1}∼{\dot{g}}_{2}}\left(w_{{\dot{g}}_{1}}\times w_{{\dot{g}}_{2}}\right)\left(w_{{\dot{g}}_{1}}+w_{{\dot{g}}_{2}}\right)$$

The notion of atom-bond connectivity index and sum connectivity index gathered by Ali et al., and initiated the new molecular descriptor named as the atom-bond sum-connectivity index in [40]:

(8) $$\;ABS\left(T,s\right)=\sum_{{\dot{g}}_{1}∼{\dot{g}}_{2}}s^{\sqrt{\frac{\left(w_{{\dot{g}}_{1}}+w_{{\dot{g}}_{2}}-2\right)}{\left(w_{{\dot{g}}_{1}}+w_{{\dot{g}}_{2}}\right)}}}\;\;ABS=\sum_{{\dot{g}}_{1}∼{\dot{g}}_{2}}\sqrt{\frac{\left(w_{{\dot{g}}_{1}}+w_{{\dot{g}}_{2}}-2\right)}{\left(w_{{\dot{g}}_{1}}+w_{{\dot{g}}_{2}}\right)}}$$

The idea of entropy was initiated by Shannon in 1948 [41]. The quantity of thermal energy per unit temperature in a system that is not accessible for meaningful work is measured by entropy [42,43]. The system’s molecular disorder is also measured by Entropy [44,45]. In this article, we have computed entropies of metal organic frameworks of T(g,h) [46,47,48].

## 2. Entropy Measures

The entropy measure of edge-weighted graph was initiated in 2009 [49], $T=(\left(V_{T},E_{T}\right),\psi \left(w_{{\dot{g}}_{1}}w_{{\dot{g}}_{2}}\right))$ for an edge-weighted graph, where $V_{T}$ is the vertex set, $E_{T}$ the edge set, and the edge-weight of an edge $\left(w_{{\dot{g}}_{1}}w_{{\dot{g}}_{2}}\right)$ is represented by $\psi (w_{{\dot{g}}_{1}}w_{{\dot{g}}_{2}})$. The entropy of a graph *T* is

(9) $$ENT_{\psi (T)}=-\sum_{{\dot{g}}_{1}∼{\dot{g}}_{2}}\frac{\psi (w_{{\dot{g}}_{1}}w_{{\dot{g}}_{2}})}{\sum_{{\dot{g}}_{1}∼{\dot{g}}_{2}}\psi \left(w_{{\dot{g}}_{1}}w_{{\dot{g}}_{2}}\right)}\log\{\frac{\psi (w_{{\dot{g}}_{1}}w_{{\dot{g}}_{2}})}{\sum_{{\dot{g}}_{1}∼{\dot{g}}_{2}}\psi \left(w_{{\dot{g}}_{1}}w_{{\dot{g}}_{2}}\right)}\}.$$

- **The first K-Banhatti entropy**
  Let $\psi \left(w_{{\dot{g}}_{1}}w_{{\dot{g}}_{2}}\right)=w_{{\dot{g}}_{1}}+w_{{\dot{g}}_{2}}$. The first *K*-Banhatti index (1) is
  $$\begin{array}{ccc} B_{1}\left(T\right) & = & \sum_{{\dot{g}}_{1}∼{\dot{g}}_{2}}\{w_{{\dot{g}}_{1}}+w_{{\dot{g}}_{2}}\}=\sum_{{\dot{g}}_{1}∼{\dot{g}}_{2}}\psi \left(w_{{\dot{g}}_{1}}w_{{\dot{g}}_{2}}\right). \end{array}$$
  The first *K*-Banhatti entropy is obtained using Equation (9)
  (10) $$ENT_{B_{1}\left(T\right)}=\log\left(B_{1}\left(T\right)\right)-\frac{1}{B_{1}\left(T\right)}\log\{{\prod_{{\dot{g}}_{1}∼{\dot{g}}_{2}}\left[w_{{\dot{g}}_{1}}+w_{{\dot{g}}_{2}}\right]}^{\left[w_{{\dot{g}}_{1}}+w_{{\dot{g}}_{2}}\right]}\}.$$
- **The second K-Banhatti entropy**
  Let $\psi \left(w_{{\dot{g}}_{1}}w_{{\dot{g}}_{2}}\right)=w_{{\dot{g}}_{1}}\times w_{{\dot{g}}_{2}}$. The second *K*-Banhatti index (2) is
  $$\begin{array}{c} B_{2}\left(T\right)=\sum_{{\dot{g}}_{1}∼{\dot{g}}_{2}}\{\left(w_{{\dot{g}}_{1}}\times w_{{\dot{g}}_{2}}\right)\}=\sum_{{\dot{g}}_{1}∼{\dot{g}}_{2}}\psi \left(w_{{\dot{g}}_{1}}w_{{\dot{g}}_{2}}\right). \end{array}$$
  The second *K*-Banhatti entropy is obtained using Equation (9)
  (11) $$ENT_{B_{2}\left(T\right)}=\log\left(B_{2}\left(T\right)\right)-\frac{1}{B_{2}\left(T\right)}\log\{{\prod_{{\dot{g}}_{1}∼{\dot{g}}_{2}}\left[w_{{\dot{g}}_{1}}\times w_{{\dot{g}}_{2}}\right]}^{\left[w_{{\dot{g}}_{1}}\times w_{{\dot{g}}_{2}}\right]}\}.$$
- **The first *K*-hyper Banhatti entropy**
  Let $\psi \left(w_{{\dot{g}}_{1}}w_{{\dot{g}}_{2}}\right)={\left(w_{{\dot{g}}_{1}}+w_{{\dot{g}}_{2}}\right)}^{2}$. The second *K*-hyper Banhatti index (3) is
  $$\begin{array}{c} HB_{1}\left(T\right)=\sum_{{\dot{g}}_{1}∼{\dot{g}}_{2}}\{{\left(w_{{\dot{g}}_{1}}+w_{{\dot{g}}_{2}}\right)}^{2}\}=\sum_{{\dot{g}}_{1}∼{\dot{g}}_{2}}\psi \left(w_{{\dot{g}}_{1}}w_{{\dot{g}}_{2}}\right). \end{array}$$
  The first *K*-hyper Banhatti entropy is obtained using Equation (9)
  (12) $$ENT_{HB_{1}\left(T\right)}=\log\left(HB_{1}\left(T\right)\right)-\frac{1}{HB_{1}\left(T\right)}\log\{{\prod_{{\dot{g}}_{1}∼{\dot{g}}_{2}}\left[w_{{\dot{g}}_{1}}+w_{{\dot{g}}_{2}}\right]}^{2{\left[w_{{\dot{g}}_{1}}+w_{{\dot{g}}_{2}}\right]}^{2}}\}.$$
- **The second K-hyper Banhatti entropy**
  Let $\psi \left(w_{{\dot{g}}_{1}}w_{{\dot{g}}_{2}}\right)={\left(w_{{\dot{g}}_{1}}\times w_{{\dot{g}}_{2}}\right)}^{2}$. The second *K*-hyper Banhatti index (4) is
  $$\begin{array}{ccc} HB_{2}\left(T\right) & = & \sum_{{\dot{g}}_{1}∼{\dot{g}}_{2}}\{{\left(w_{{\dot{g}}_{1}}\times w_{{\dot{g}}_{2}}\right)}^{2}\}=\sum_{{\dot{g}}_{1}∼{\dot{g}}_{2}}\psi \left(w_{{\dot{g}}_{1}}w_{{\dot{g}}_{2}}\right). \end{array}$$
  The second *K*-hyper Banhatti entropy is obtained using Equation (9)
  (13) $$ENT_{HB_{2}\left(T\right)}=\log\left(HB_{1}\left(T\right)\right)-\frac{1}{HB_{1}\left(T\right)}\log\{{\prod_{{\dot{g}}_{1}∼{\dot{g}}_{2}}\left[w_{{\dot{g}}_{1}}\times w_{{\dot{g}}_{2}}\right]}^{2{\left[w_{{\dot{g}}_{1}}\times w_{{\dot{g}}_{2}}\right]}^{2}}\}.$$
- **The first redefined Zagreb entropy**
  Let $\psi \left(w_{{\dot{g}}_{1}}w_{{\dot{g}}_{2}}\right)=\frac{w_{{\dot{g}}_{1}}+w_{{\dot{g}}_{2}}}{w_{{\dot{g}}_{1}}w_{{\dot{g}}_{2}}}$. The first redefined Zagreb index (5) is
  $$\begin{array}{ccc} ReZG_{1} & = & \sum_{{\dot{g}}_{1}∼{\dot{g}}_{2}}\{\frac{w_{{\dot{g}}_{1}}+w_{{\dot{g}}_{2}}}{w_{{\dot{g}}_{1}}w_{{\dot{g}}_{2}}}\}=\sum_{{\dot{g}}_{1}∼{\dot{g}}_{2}}\psi \left(w_{{\dot{g}}_{1}}w_{{\dot{g}}_{2}}\right). \end{array}$$
  The first redefined Zagreb entropy is obtained using Equation (9)
  (14) $$ENT_{ReZG_{1}}=\log\left(ReZG_{1}\right)-\frac{1}{ReZG_{1}}\log\{{\prod_{{\dot{g}}_{1}∼{\dot{g}}_{2}}\left[\frac{w_{{\dot{g}}_{1}}+w_{{\dot{g}}_{2}}}{w_{{\dot{g}}_{1}}w_{{\dot{g}}_{2}}}\right]}^{\left[\frac{w_{{\dot{g}}_{1}}+w_{{\dot{g}}_{2}}}{w_{{\dot{g}}_{1}}w_{{\dot{g}}_{2}}}\right]}\}.$$
- **The second redefined Zagreb entropy**
  Let $\psi \left(w_{{\dot{g}}_{1}}w_{{\dot{g}}_{2}}\right)=\frac{w_{{\dot{g}}_{1}}d_{v}}{w_{{\dot{g}}_{1}}+w_{{\dot{g}}_{2}}}$. The second redefined Zagreb index (6) is
  $$\begin{array}{ccc} ReZG_{2} & = & \sum_{{\dot{g}}_{1}∼{\dot{g}}_{2}}\{\frac{w_{{\dot{g}}_{1}}w_{{\dot{g}}_{2}}}{w_{{\dot{g}}_{1}}+w_{{\dot{g}}_{2}}}\}=\sum_{{\dot{g}}_{1}∼{\dot{g}}_{2}}\psi \left(w_{{\dot{g}}_{1}}w_{{\dot{g}}_{2}}\right). \end{array}$$
  The second redefined Zagreb entropy is obtained using Equation (9)
  (15) $$ENT_{ReZG_{2}}=\log\left(ReZG_{2}\right)-\frac{1}{ReZG_{2}}\log\{{\prod_{{\dot{g}}_{1}∼{\dot{g}}_{2}}\left[\frac{w_{{\dot{g}}_{1}}d_{v}}{w_{{\dot{g}}_{1}}+w_{{\dot{g}}_{2}}}\right]}^{\left[\frac{w_{{\dot{g}}_{1}}w_{{\dot{g}}_{2}}}{w_{{\dot{g}}_{1}}+w_{{\dot{g}}_{2}}}\right]}\}.$$
- **The third redefined Zagreb entropy**
  Let $\psi \left(w_{{\dot{g}}_{1}}w_{{\dot{g}}_{2}}\right)=\{\left(w_{{\dot{g}}_{1}}w_{{\dot{g}}_{2}}\right)\left(w_{{\dot{g}}_{1}}+w_{{\dot{g}}_{2}}\right)\}$. The third redefined Zagreb index (7) is
  $$\begin{array}{ccc} ReZG_{3} & = & \sum_{{\dot{g}}_{1}∼{\dot{g}}_{2}}\{\left(w_{{\dot{g}}_{1}}w_{{\dot{g}}_{2}}\right)\left(d_{g_{1}}+d_{g_{2}}\right)\}=\sum_{{\dot{g}}_{1}∼{\dot{g}}_{2}}\psi \left(w_{{\dot{g}}_{1}}w_{{\dot{g}}_{2}}\right). \end{array}$$
  The third redefined Zagreb entropy is obtained by using Equation (9)
  (16) $$ENT_{ReZG_{3}}=\log\left(ReZG_{3}\right)-\frac{1}{ReZG_{3}}\log\{{\prod_{{\dot{g}}_{1}∼{\dot{g}}_{2}}\left[\left(w_{{\dot{g}}_{1}}w_{{\dot{g}}_{2}}\right)\left(w_{{\dot{g}}_{1}}+w_{{\dot{g}}_{2}}\right)\right]}^{\left[\left(w_{{\dot{g}}_{1}}w_{{\dot{g}}_{2}}\right)\left(w_{{\dot{g}}_{1}}+w_{{\dot{g}}_{2}}\right)\right]}\}.$$
- **Atom-bond sum connectivity Entropy**
  Let $\psi \left({\dot{g}}_{1}{\dot{g}}_{2}\right)=\{\sqrt{\frac{w_{{\dot{g}}_{1}}+w_{{\dot{g}}_{2}}-2}{w_{{\dot{g}}_{1}}+w_{{\dot{g}}_{2}}}}\}$. The atom-bond connectivity index (8) is
  $$\begin{array}{ccc} ABS(T) & = & \sum_{{\dot{g}}_{1},{\dot{g}}_{2}\in E_{T}}^{}\{\sqrt{\frac{w_{{\dot{g}}_{1}}+w_{{\dot{g}}_{2}}-2}{w_{{\dot{g}}_{1}}+w_{{\dot{g}}_{2}}}}\}=\sum_{{\dot{g}}_{1},{\dot{g}}_{2}\in E_{G}}^{}\psi \left({\dot{g}}_{1}{\dot{g}}_{2}\right). \end{array}$$
  The atom-bond sum connectivity $\left(ENT_{ABC(T)}\right)$ entropy is obtained using Equation (9)
  (17) $$ENT_{ABS(T)}=\log\left(ABS(T)\right)-\frac{1}{ABS(T)}\log\{{\prod_{{\dot{g}}_{1},{\dot{g}}_{2}\in E_{T}}\left(\sqrt{\frac{w_{{\dot{g}}_{1}}+w_{{\dot{g}}_{2}}-2}{w_{{\dot{g}}_{1}}+w_{{\dot{g}}_{2}}}}\right)}^{\left(\sqrt{\frac{w_{{\dot{g}}_{1}}+w_{{\dot{g}}_{2}}-2}{w_{{\dot{g}}_{1}}+w_{{\dot{g}}_{2}}}}\right)}\}.$$

## 3. Entropy Measure of FeTPyP-Co T(g,h)

**Figure 1:**
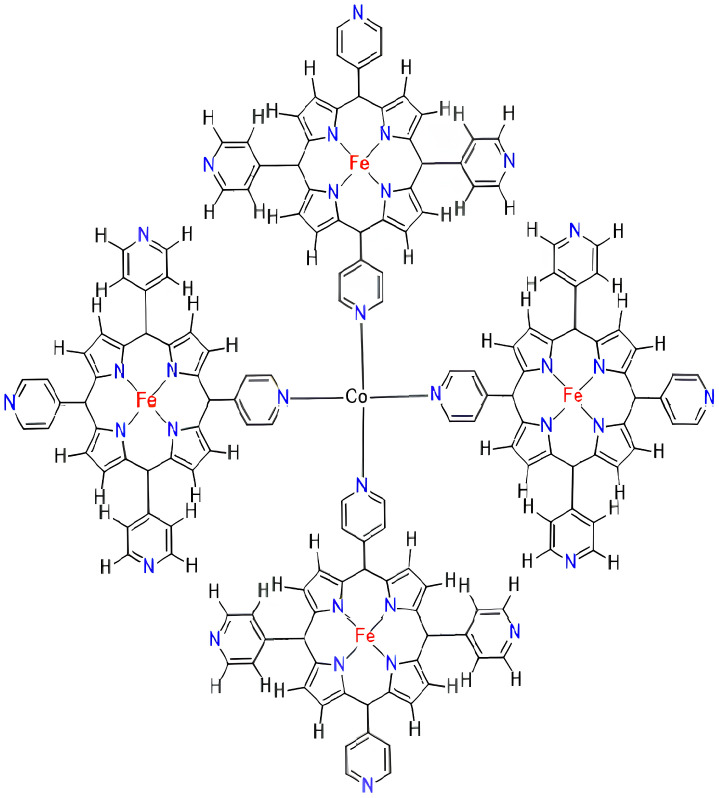
FeTPyP-Co MOFs Structure.

The FeTPyP-Co MOFs, also known as iron(III) tetra-p-tolyl porphyrin (FeTPyP) frameworks coordinated with cobalt (Co) ligands, are a type of molecular organic framework. The structure of FeTPyP-Co MOFs consist of a central iron(III)ion coordinated with four p-tolylporphyrin (TPyP) ligands and one Co ligand. The TPyP ligands provide a tetradentate coordination, while the Co ligand provides a monodentate coordination. The properties of FeTPyP-Co MOFs exhibit catalytic activity for a variety of reactions, including oxidation reactions and cyclohexane oxidation. The Co ligand can modulate the redox properties of the iron center, enhancing its ability to oxidize substrates [50]. FeTPyP-Co MOFs have been studied for their magnetic properties, which are influenced by the coordination environment of the iron center. The TPyP ligands can induce antiferromagnetic coupling between the iron centers, while the Co ligand can modulate the magnitude of the coupling. FeTPyP-Co MOFs have also been investigated for their optical properties, which arise from the TPyP ligands. The TPyP ligands can absorb visible light and undergo photoinduced electron transfer, leading to the generation of reactive intermediates with potential applications in photocatalysis. Overall, FeTPyP-Co MOFs are a promising class of molecular organic frameworks with diverse applications in catalysis, electrocatalysis, magnetism, and optics. T(g,h) is a graph of FeTPyP-Co (TPyP ¼ Tetrakis pyridyl porphyrin) metal–organic frameworks, which embodies cells in rows and embodies cells in columns. The molecular graph of FeTPyP-Co is given in Figure 1. There are total 74gh vertices and 88gh−2g−2h+1 edges. In this article, we tried to explain T(g,h), with a total atom count of 74gh; as described in Figure 1.

Table 1 represents the atom-bond partitions of T(g,h) derived from these results.

- **The first K-Banhatti entropy measure of T(g,h)**
  Table 1 and Equation (1) imply:
  (18) $$\begin{array}{ccc} B_{1}\left(T\left(g,h\right),s\right) & = & \sum_{\left(1,3\right)}s^{1+3}+\sum_{\left(2,3\right)}s^{2+3}+\sum_{\left(3,3\right)}s^{3+3}+\sum_{\left(3,4\right)}s^{3+4}\;\\ \; & = & \left(24gh+1\right)s^{4}+\left(6g+6h-6\right)s^{5}+\left(56gh-4g-4h+2\right)s^{6}\;\\ \; & + & \left(8gh-4g-4h+4\right)x^{7}. \end{array}$$
  After differentiating Equation (18), we obtain the first *K*-Banhatti index at s=1.
  (19) $$B_{1}\left(T\left(g,h\right)\right)=488gh-82g-22h+10.$$
  The first *K*-Banhatti entropy measure of T(g,h) is obtained using Equation (19) and Table 1 in Equation (10):
  $$\begin{array}{ccc} ENT_{B_{1}}\left(T\left(g,h\right)\right) & = & \log\left(B_{1}\right)-\frac{1}{B_{1}}\log\{{\prod_{E_{\left(1,3\right)}}\left(w_{{\dot{g}}_{1}}+w_{{\dot{g}}_{2}}\right)}^{\left(w_{{\dot{g}}_{1}}+w_{{\dot{g}}_{2}}\right)}\times {\prod_{E_{\left(2,3\right)}}\left(w_{{\dot{g}}_{1}}+w_{{\dot{g}}_{2}}\right)}^{\left(w_{{\dot{g}}_{1}}+w_{{\dot{g}}_{2}}\right)}\;\\ & \times & {\prod_{E_{\left(3,3\right)}}\left(w_{{\dot{g}}_{1}}+w_{{\dot{g}}_{2}}\right)}^{\left(w_{{\dot{g}}_{1}}+w_{{\dot{g}}_{2}}\right)}\times {\prod_{E_{\left(3,4\right)}}\left(w_{{\dot{g}}_{1}}+w_{{\dot{g}}_{2}}\right)}^{\left(w_{{\dot{g}}_{1}}+w_{{\dot{g}}_{2}}\right)}\;\\ & = & \log\left(488gh-82g-22h+10\right)-\frac{1}{488gh-82g-22h+10}\log\{\left(24gh+1\right){\left(4\right)}^{4}\;\\ & \times & \left(6g+6h-6\right){\left(5\right)}^{5}\times \left(56gh-4g-4h+2\right){\left(6\right)}^{6}\times \left(8gh-4g-4h+4\right){\left(7\right)}^{7}. \end{array}$$
- **The second *K*-Banhatti entropy measure of T(g,h)**
  In view of Table 1 and Equation (2), we obtain
  (20) $$\begin{array}{ccc} B_{2}\left(T\left(g,h\right)\right) & = & \sum_{\left(1,3\right)}s^{1\times 3}+\sum_{\left(2,3\right)}s^{2\times 3}+\sum_{\left(3,3\right)}s^{3\times 3}+\sum_{\left(3,4\right)}s^{3\times 4}\;\\ \; & = & \left(24gh+1\right)s^{3}+\left(6g+6h-6\right)s^{6}+\left(56gh-4h-4f+2\right)s^{9}\;\\ \; & + & \left(8gh-4e-4f+2\right)s^{12}. \end{array}$$
  After differentiating Equation (20) at s=1, we obtain the second *K*-Banhatti index
  (21) $$B_{2}\left(T\left(g,h\right)\right)=744gh-48g-48h+6.$$
  The second *K*-Banhatti entropy measure of T(g,h) is obtained in view of Equation (21), Table 1 and Equation (11):
  $$\begin{array}{ccc} ENT_{B_{2}}\left(T\left(g,h\right)\right) & = & \log\left(B_{2}\right)-\frac{1}{B_{2}}\log\{{\prod_{E_{\left(1,3\right)}}\left(w_{{\dot{g}}_{1}}\times w_{{\dot{g}}_{2}}\right)}^{\left(w_{{\dot{g}}_{1}}\times w_{{\dot{g}}_{2}}\right)}\times {\prod_{E_{\left(2,3\right)}}\left(w_{{\dot{g}}_{1}}\times w_{{\dot{g}}_{2}}\right)}^{\left(w_{{\dot{g}}_{1}}\times w_{{\dot{g}}_{2}}\right)}\;\\ \; & \times & {\prod_{E_{\left(3,3\right)}}\left(w_{{\dot{g}}_{1}}\times w_{{\dot{g}}_{2}}\right)}^{\left(w_{{\dot{g}}_{1}}\times w_{{\dot{g}}_{2}}\right)}\times {\prod_{E_{\left(3,4\right)}}\left(w_{{\dot{g}}_{1}}\times w_{{\dot{g}}_{2}}\right)}^{\left(w_{{\dot{g}}_{1}}\times w_{{\dot{g}}_{2}}\right)}\}\;\\ \; & = & \log\left(744gh-48g-48h+6\right)-\frac{1}{744gh-48g-48h+6}\log\{\left(24gh+1\right)\left(3^{3}\right)\;\\ \; & \times & \left(6g+6h-6\right)6^{6}\times \left(56gh-4g-4h+2\right)9^{9}\times \left(8gh-4e-4f+4\right)12^{12}\}. \end{array}$$
- **The first *K*-hyper Banhatti entropy measure of T(g,h)**
  The Equation (3) and Table 1 gives:
  (22) $$\begin{array}{ccc} HB_{1}\left(T\left(g,h\right)\right) & = & \sum_{\left(1,3\right)}s^{{\left(1+3\right)}^{2}}+\sum_{\left(2,3\right)}s^{{\left(2+3\right)}^{2}}+\sum_{\left(3,3\right)}s^{{\left(3+3\right)}^{2}}+\sum_{\left(3,4\right)}s^{{\left(3+4\right)}^{2}}\;\\ \; & = & \left(24gh+1\right)s^{16}+\left(6g+6h-6\right)s^{36}+\left(56gh-4g-4h+2\right)s^{81}\;\\ \; & + & \left(8gh-4g-4h+4\right)s^{144}. \end{array}$$
  After differentiating Equation (22) at s=1, we obtain the first *K*-hyper Banhatti index:
  (23) $$HB_{1}\left(T\left(g,h\right)\right)=6072gh-684g-684h+522.$$
  The first *K*-hyper Banhatti entropy measure of T(g,h) is obtained in view of Equation (23), Table 1, and Equation (13):
  $$\begin{array}{ccc} ENT_{HB_{1}}\left(T\left(g,h\right)\right) & = & \log\left(HB_{1}\right)-\frac{1}{HB_{1}}\log\{{\prod_{E_{\left(1,3\right)}}\left(w_{{\dot{g}}_{1}}+w_{{\dot{g}}_{2}}\right)}^{2{\left(w_{{\dot{g}}_{1}}+w_{{\dot{g}}_{2}}\right)}^{2}}\times {\prod_{E_{\left(2,3\right)}}\left(w_{{\dot{g}}_{1}}+w_{{\dot{g}}_{2}}\right)}^{2{\left(w_{{\dot{g}}_{1}}+w_{{\dot{a}}_{2}}\right)}^{2}}\;\\ \; & \times & {\prod_{E_{\left(3,3\right)}}\left(w_{{\dot{g}}_{1}}+w_{{\dot{g}}_{2}}\right)}^{2{\left(w_{{\dot{g}}_{1}}+w_{{\dot{g}}_{2}}\right)}^{2}}\times {\prod_{E_{\left(3,4\right)}}\left(w_{{\dot{g}}_{1}}+w_{{\dot{g}}_{2}}\right)}^{2{\left(w_{{\dot{g}}_{1}}+w_{{\dot{g}}_{2}}\right)}^{2}}\;\\ \; & = & \log\left(6072gh-684g-684h+522\right)-\frac{1}{6072gh-684g-684h+522}\log\{\left(24gh+1\right)\left(4^{32}\right)\;\\ \; & \times & \left(6g+6h-6\right)\left(5^{50}\right)\times \left(56gh-4g-4h+2\right)\left(6^{72}\right)\times \left(8gh-4g-4h+4\right)\left(7^{98}\right)\}. \end{array}$$
- **The second K-hyper Banhatti entropy measure of T(g,h)**
  In view of Table 1 and Equation (4), we obtain:
  (24) $$\begin{array}{ccc} HB_{2}\left(T\left(g,h\right)\right) & = & \sum_{\left(1,3\right)}s^{{\left(1\times 3\right)}^{2}}+\sum_{\left(2,3\right)}s^{{\left(2\times 3\right)}^{2}}+\sum_{\left(3,3\right)}s^{{\left(3\times 3\right)}^{2}}+\sum_{\left(3,4\right)}s^{{\left(3\times 4\right)}^{2}}\;\\ \; & = & \left(24gh+1\right)s^{9}+\left(6g+6h-6\right)s^{36}+\left(56gh-4g-4h+2\right)s^{81}\;\\ \; & + & \left(8gh-4g-4h+4\right)s^{144}. \end{array}$$
  After differentiating Equation (24) at s=1, we obtain the second *K*-hyper Banhatti index:
  (25) $$HB_{2}\left(T\left(g,h\right)\right)=5904gh-684g-684h-621.$$
  The second *K*-hyper Banhatti entropy measure of T(g,h) is obtained in view of Equation (25), Table 1, and Equation (13):
  $$\begin{array}{ccc} ENT_{HB_{1}}\left(T\left(g,h\right)\right) & = & \log\left(HB_{1}\right)-\frac{1}{HB_{1}}\log\{{\prod_{E_{\left(1,3\right)}}\left(w_{{\dot{g}}_{1}}\times w_{{\dot{g}}_{2}}\right)}^{2{\left(w_{{\dot{g}}_{1}}\times w_{{\dot{g}}_{2}}\right)}^{2}}\times {\prod_{E_{\left(2,3\right)}}\left(w_{{\dot{g}}_{1}}\times w_{{\dot{g}}_{2}}\right)}^{2{\left(w_{{\dot{g}}_{1}}\times w_{{\dot{g}}_{2}}\right)}^{2}}\;\\ \; & \times & {\prod_{E_{\left(3,3\right)}}\left(w_{{\dot{g}}_{1}}\times w_{{\dot{g}}_{2}}\right)}^{2{\left(w_{{\dot{g}}_{1}}\times w_{{\dot{g}}_{2}}\right)}^{2}}\times {\prod_{E_{\left(3,4\right)}}\left(w_{{\dot{g}}_{1}}\times w_{{\dot{g}}_{2}}\right)}^{2{\left(w_{{\dot{g}}_{1}}\times w_{{\dot{g}}_{2}}\right)}^{2}}\;\\ \; & = & \log\left(5904gh-684g-684h-621\right)-\frac{1}{5904gh-684g-684h-621}\log\{\left(24gh+1\right){\left(3\right)}^{18}\;\\ \; & \times & \left(6g+6h-6\right)6^{72}\times \left(56gh-4g-4h+2\right)9^{162}\times \left(8gh-4g-4h+4\right)12^{288}\}. \end{array}$$
- **The first redefined Zagreb entropy measure of T(g,h)**
  Using Equation (5) and Table 1, we get:
  (26) $$\begin{array}{ccc} ReZG_{1}\left(T\left(g,h\right)\right) & = & \sum_{\left(1,3\right)}s^{\frac{1+3}{1\times 3}}+\sum_{\left(2,3\right)}s^{\frac{2+3}{2\times 3}}+\sum_{\left(3,3\right)}s^{\frac{3+3}{3\times 3}}+\sum_{\left(3,4\right)}s^{\frac{3+4}{3\times 4}}\;\\ \; & = & \left(24gh+1\right)s^{\frac{4}{3}}+\left(6g+6h-6\right)s^{\frac{5}{6}}+\left(56gh-4g-4h+4\right)s^{\frac{6}{9}}\;\\ \; & + & \left(8gh-4g-4h+4\right)s^{\frac{7}{12}}. \end{array}$$
  After differentiating Equation (26) at s=1, we obtain
  (27) $$ReZG_{1}\left(T\left(g,h\right)\right)=74gh-\frac{4}{3}.$$
  The first redefined Zagreb entropy measure is obtained in view of Equation (27), Table 1, and Equation (14):
  $$\begin{array}{ccc} ENT_{ReZG_{1}}\left(T\left(g,h\right)\right) & = & \log\left(ReZG_{1}\right)-\frac{1}{ReZG_{1}}\log\{{\prod_{E_{\left(1,3\right)}}\left[\frac{w_{{\dot{g}}_{1}}+w_{{\dot{g}}_{2}}}{w_{{\dot{g}}_{1}}w_{{\dot{g}}_{2}}}\right]}^{\left[\frac{w_{{\dot{g}}_{1}}+w_{{\dot{g}}_{2}}}{w_{{\dot{g}}_{1}}d_{v}}\right]}\;\\ \; & \times & {\prod_{E_{\left(2,3\right)}}\left[\frac{w_{{\dot{g}}_{1}}+w_{{\dot{g}}_{2}}}{w_{{\dot{g}}_{1}}w_{{\dot{g}}_{2}}}\right]}^{\left[\frac{w_{{\dot{g}}_{1}}+d_{v}}{w_{{\dot{g}}_{1}}w_{{\dot{g}}_{2}}}\right]}\times {\prod_{E_{\left(3,3\right)}}\left[\frac{w_{{\dot{g}}_{1}}+w_{{\dot{g}}_{2}}}{w_{{\dot{g}}_{1}}w_{{\dot{g}}_{2}}}\right]}^{\left[\frac{w_{{\dot{g}}_{1}}+w_{{\dot{g}}_{2}}}{w_{{\dot{g}}_{1}}w_{{\dot{g}}_{2}}}\right]}\;\\ \; & \times & {\prod_{E_{\left(3,4\right)}}\left[\frac{w_{{\dot{g}}_{1}}+w_{{\dot{g}}_{2}}}{w_{{\dot{g}}_{1}}w_{{\dot{g}}_{2}}}\right]}^{\left[\frac{w_{{\dot{g}}_{1}}+d_{v}}{w_{{\dot{g}}_{1}}w_{{\dot{g}}_{2}}}\right]}\}\;\\ \; & = & \log\left(74gh-\frac{4}{3}\right)\;\\ \; & - & \frac{1}{\left(74gh-\frac{4}{3}\right)}\log\{\left(24gh+1\right){\left(\frac{4}{3}\right)}^{\frac{4}{3}}\;\\ \; & \times & \left(6g+6h-6\right){\left(\frac{5}{6}\right)}^{\frac{5}{6}}\;\\ \; & \times & \left(56gh-4g-4h+2\right){\left(\frac{6}{9}\right)}^{\frac{6}{9}}\times \left(8gh-4g-4h+4\right){\left(\frac{7}{12}\right)}^{\frac{7}{12}}\}. \end{array}$$
- **The second redefined Zagreb entropy measure of T(g,h)**
  In view of Table 1 and Equation (6), we have:
  (28) $$\begin{array}{ccc} ReZG_{2}\left(T\left(g,h\right)\right) & = & \sum_{\left(1,3\right)}s^{\frac{1\times 3}{1+3}}+\sum_{\left(2,3\right)}s^{\frac{2\times 3}{2+3}}+\sum_{\left(3,3\right)}s^{\frac{3\times 3}{3+3}}+\sum_{\left(3,4\right)}s^{\frac{3\times 4}{3+4}}\;\\ \; & = & \left(24gh+1\right)s^{\frac{3}{4}}+\left(6g+6h-6\right)s^{\frac{6}{5}}+\left(56gh-4g-4h+2\right)s^{\frac{9}{6}}\;\\ \; & + & \left(8gh-4g-4h+4\right)s^{\frac{12}{7}}. \end{array}$$
  After differentiating Equation (28) at s=1, we obtain
  (29) $$ReZG_{2}\left(T\left(g,h\right)\right)=\frac{810}{7}gh-\frac{198}{35}g-\frac{198}{35}h-\frac{117}{35}.$$
  The second redefined Zagreb entropy measure is obtained in view of Equation (29), Table 1, and Equation (15):
  $$\begin{array}{ccc} ENT_{ReZG_{2}}\left(T\left(g,h\right)\right)\; & = & \log\left(ReZG_{2}\right)-\frac{1}{ReZG_{2}}\log\{{\prod_{E_{\left(1,3\right)}}\left[\frac{w_{{\dot{g}}_{1}}w_{{\dot{g}}_{2}}}{w_{{\dot{g}}_{1}}+w_{{\dot{g}}_{2}}}\right]}^{\left[\frac{w_{{\dot{g}}_{1}}w_{{\dot{g}}_{2}}}{w_{{\dot{g}}_{1}}+w_{{\dot{g}}_{2}}}\right]}\;\\ \times {\prod_{E_{\left(2,3\right)}}\left[\frac{w_{{\dot{g}}_{1}}w_{{\dot{g}}_{2}}}{w_{{\dot{g}}_{1}}+w_{{\dot{g}}_{2}}}\right]}^{\left[\frac{w_{{\dot{g}}_{1}}w_{{\dot{g}}_{2}}}{d_{u}+w_{{\dot{g}}_{2}}}\right]}\; & \times & {\prod_{E_{\left(3,3\right)}}\left[\frac{w_{{\dot{g}}_{1}}w_{{\dot{g}}_{2}}}{w_{{\dot{g}}_{1}}+w_{{\dot{g}}_{2}}}\right]}^{\left[\frac{w_{{\dot{g}}_{1}}w_{{\dot{g}}_{2}}}{w_{{\dot{g}}_{1}}+w_{{\dot{g}}_{2}}}\right]}\times {\prod_{E_{\left(3,4\right)}}\left[\frac{w_{{\dot{g}}_{1}}w_{{\dot{g}}_{2}}}{w_{{\dot{g}}_{1}}+w_{{\dot{g}}_{2}}}\right]}^{\left[\frac{w_{{\dot{g}}_{1}}w_{{\dot{g}}_{2}}}{w_{{\dot{g}}_{1}}+w_{{\dot{g}}_{2}}}\right]}\}\;\\ \; & = & \log\left(\frac{810}{7}gh-\frac{198}{35}g-\frac{198}{35}h-\frac{117}{35}\right)\;\\ \; & - & \frac{1}{\frac{810}{7}gh-\frac{198}{35}g-\frac{198}{35}h-\frac{117}{35}}\log\{\left(24gh+1\right){\left(\frac{3}{4}\right)}^{\frac{3}{4}}\;\\ \; & \times & \left(6g+6h-6\right){\left(\frac{6}{5}\right)}^{\frac{6}{5}}\times \left(56gh-4g-4h+2\right){\left(\frac{9}{6}\right)}^{\frac{9}{6}}\;\\ \; & \times & \left(8gh-4g-4h+4\right){\left(\frac{12}{7}\right)}^{\frac{12}{7}}\}. \end{array}$$
- **The third redefined Zagreb entropy measure of T(g,h)**
  The Table 1 and Equation (7) implies:
  (30) $$\begin{array}{ccc} ReZG_{3}\left(T\left(g,h\right)\right) & = & \sum_{\left(1,3\right)}s^{\left(1\times 3)(1+3\right)}+\sum_{\left(2,3\right)}s^{\left(2\times 3)(2+3\right)}+\sum_{\left(3,3\right)}s^{\left(3\times 3)(3+3\right)}+\sum_{\left(3,4\right)}s^{\left(3\times 4)(3+4\right)}\;\\ \; & = & \left(24gh+1\right)s^{12}+\left(6g+6h-6\right)s^{30}+\left(56gh-4g-4h+2\right)s^{54}\;\\ \; & + & \left(8gh-4g-4h+4\right)s^{84}. \end{array}$$
  After differentiating Equation (30) at s=1, we get
  (31) $$ReZG_{3}\left(T\left(g,h\right)\right)=3984gh-534g-534h+270.$$
  The third redefined Zagreb entropy measure is obtained in view of Equation (31), Table 1, and Equation (16):
  $$\begin{array}{ccc} ENT_{ReZG_{3}}\left(T\left(g,h\right)\right) & = & \log\left(ReZG_{3}\right)-\frac{1}{ReZG_{3}}\log\{{\prod_{E_{\left(1,3\right)}}\left[\left(d_{u}w_{{\dot{g}}_{2}}\right)\left(d_{u}+w_{{\dot{g}}_{2}}\right)\right]}^{\left[\left(w_{{\dot{g}}_{1}}w_{{\dot{g}}_{2}}\right)\left(w_{{\dot{g}}_{1}}+w_{{\dot{g}}_{2}}\right)\right]}\;\\ \; & \times & {\prod_{E_{\left(2,3\right)}}\left[\left(w_{{\dot{g}}_{1}}w_{{\dot{g}}_{2}}\right)\left(w_{{\dot{g}}_{1}}+w_{{\dot{g}}_{2}}\right)\right]}^{\left[\left(d_{u}w_{{\dot{g}}_{2}}\right)\left(w_{{\dot{g}}_{1}}+w_{{\dot{g}}_{2}}\right)\right]}\;\\ \; & \times & {\prod_{E_{\left(3,3\right)}}\left[\left(w_{{\dot{g}}_{1}}w_{{\dot{g}}_{2}}\right)\left(w_{{\dot{g}}_{1}}+w_{{\dot{g}}_{2}}\right)\right]}^{\left[\left(w_{{\dot{g}}_{1}}w_{{\dot{g}}_{2}}\right)\left(w_{{\dot{g}}_{1}}+w_{{\dot{g}}_{2}}\right)\right]}\;\\ \; & \times & {\prod_{E_{\left(3,4\right)}}\left[\left(w_{{\dot{g}}_{1}}w_{{\dot{g}}_{2}}\right)\left(w_{{\dot{g}}_{1}}+w_{{\dot{g}}_{2}}\right)\right]}^{\left[\left(w_{{\dot{g}}_{1}}w_{{\dot{g}}_{2}}\right)\left(w_{{\dot{g}}_{1}}+w_{{\dot{g}}_{2}}\right)\right]}\}\;\\ \; & = & \log\left(3984gh-534g-534h+270\right)-\frac{1}{\left(3984gh-534g-534h+270\right)}\log\{\left(24gh+1\right){\left(12\right)}^{12}\;\\ \; & \times & \left(6g+6h-6\right)30^{30}\times \left(56gh-4g-4h+2\right)54^{54}\;\\ \; & \times & \left(8gh-4g-4h+4\right)84^{84}\}. \end{array}$$
- **Atom-bond sum connectivity entropy measure of T(g,h)**
  In view of Table 1 and Equation (8), we get
  (32) $$\begin{array}{ccc} ABS(T(g,h)) & = & \sum_{\left(1,3\right)}s^{\sqrt{\frac{1+3-2}{1+3}}}+\sum_{\left(2,3\right)}s^{\sqrt{\frac{2+3-2}{2+3}}}+\sum_{\left(3,3\right)}s^{\sqrt{\frac{3+3-2}{3+3}}}+\sum_{\left(3,4\right)}s^{\sqrt{\frac{3+4-2}{3+4}}}\;\\ & = & \left(24gh+1\right)s^{\sqrt{\frac{2}{4}}}+\left(6g+6h-6\right)s^{\sqrt{\frac{3}{5}}}+\left(56gh-4g-4h+2\right)s^{\sqrt{\frac{4}{6}}}\;\\ & + & \left(8gh-4g-4h+4\right)s^{\sqrt{\frac{5}{7}}}. \end{array}$$
  After differentiating Equation (32) at s=1, we have
  (33) $$\begin{array}{ccc} ABS(T(g,h)) & = & \left(24gh+1\right)\sqrt{\frac{2}{4}}+\left(6g+6h-6\right)\sqrt{\frac{3}{5}}+\left(56gh-4g-4h+2\right)\sqrt{\frac{4}{6}}\\ & + & \left(8gh-4g-4h+4\right)\sqrt{\frac{5}{7}}. \end{array}$$
  The atom-bond sum connectivity entropy measure is obtained in view of Equation (33), Table 1, and Equation (17):
  $$\begin{array}{ccc} ENT_{ABS}\left(T\left(g,h\right)\right) & = & \log\left(ABS\right)-\frac{1}{ABS}\log\{{\prod_{E_{\left(1,3\right)}}\left[\sqrt{\frac{\left(w_{{\dot{g}}_{1}}+w_{{\dot{g}}_{2}}-2\right)}{\left(w_{{\dot{g}}_{1}}+w_{{\dot{g}}_{2}}\right)}}\right]}^{\left[\sqrt{\frac{\left(w_{{\dot{g}}_{1}}+w_{{\dot{g}}_{2}}-2\right)}{\left(w_{{\dot{g}}_{1}}+w_{{\dot{g}}_{2}}\right)}}\right]}\;\\ \; & \times & {\prod_{E_{\left(2,3\right)}}\left[\sqrt{\frac{\left(w_{{\dot{g}}_{1}}+w_{{\dot{g}}_{2}}-2\right)}{\left(w_{{\dot{g}}_{1}}+w_{{\dot{g}}_{2}}\right)}}\right]}^{\left[\sqrt{\frac{\left(w_{{\dot{g}}_{1}}+w_{{\dot{g}}_{2}}-2\right)}{\left(w_{{\dot{g}}_{1}}+w_{{\dot{g}}_{2}}\right)}}\right]}\times {\prod_{E_{\left(3,3\right)}}\left[\sqrt{\frac{\left(w_{{\dot{g}}_{1}}+w_{{\dot{g}}_{2}}-2\right)}{\left(w_{{\dot{g}}_{1}}+w_{{\dot{g}}_{2}}\right)}}\right]}^{\left[\sqrt{\frac{\left(w_{{\dot{g}}_{1}}+w_{{\dot{g}}_{2}}-2\right)}{\left(w_{{\dot{g}}_{1}}+w_{{\dot{g}}_{2}}\right)}}\right]}\;\\ \; & \times & {\prod_{E_{\left(3,4\right)}}\left[\sqrt{\frac{\left(w_{{\dot{g}}_{1}}+w_{{\dot{g}}_{2}}-2\right)}{\left(w_{{\dot{g}}_{1}}+w_{{\dot{g}}_{2}}\right)}}\right]}^{\left[\sqrt{\frac{\left(w_{{\dot{g}}_{1}}+w_{{\dot{g}}_{2}}-2\right)}{\left(w_{{\dot{g}}_{1}}+w_{{\dot{g}}_{2}}\right)}}\right]}\}\;\\ \; & = & \log\left(ABS\right)-\frac{1}{ABS}\log\{\left(24gh+1\right){\left(\sqrt{\frac{2}{4}}\right)}^{\sqrt{\frac{2}{4}}}\times \left(6g+6h-6\right){\left(\sqrt{\frac{3}{5}}\right)}^{\sqrt{\frac{3}{5}}}\;\\ \; & \times & \left(56gh-4g-4h+2\right){\left(\sqrt{\frac{4}{6}}\right)}^{\sqrt{\frac{5}{7}}}\times \left(8gh-4g-4h+4\right){\left(\sqrt{\frac{5}{7}}\right)}^{\sqrt{\frac{5}{7}}}\}. \end{array}$$

### Comparison

**Figure 2:**
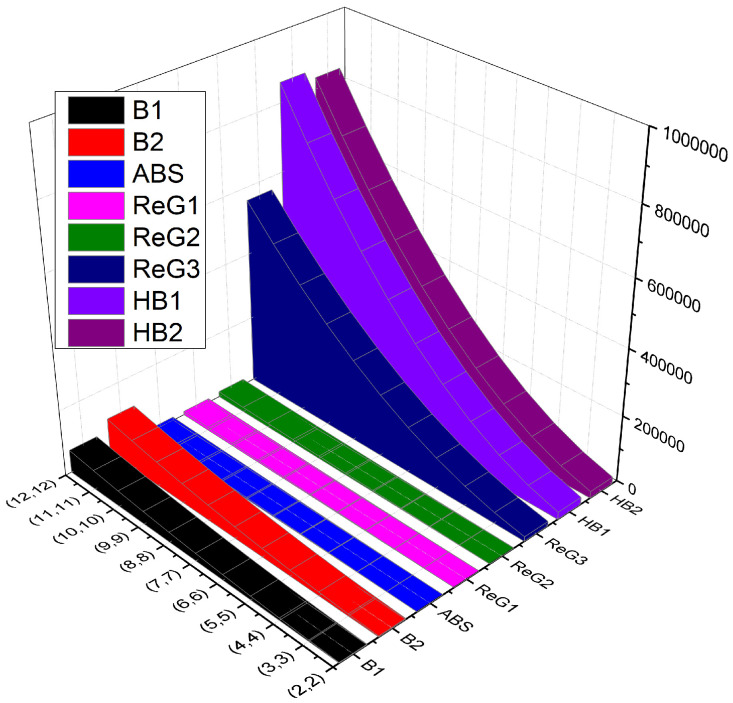
Graphical comparison of indices of T(g,h).

In this section, comparison (numerical in Table 2 and graphical in Figure 2) of various computed *K*-Banhatti and the redefined Zagreb indices is presented.

## 4. Entropy Measure of CoBHT (CO) Lattice

The CoBHT (CO) lattice refers to a type of molecular organic framework in which cobalt (Co) is coordinated with 2,3,5,6-tetrafluoro-7,7,8,8-tetracyanoquinodimethane (TCNQ) ligands and carbon monoxide (CO) ligands. The structure of the CoBHT (CO) lattice consists of a one-dimensional array of Co atoms coordinated with TCNQ and CO ligands. Each Co atom is coordinated with four TCNQ ligands and two CO ligands, forming an octahedral coordination geometry. The TCNQ ligands stack along the one-dimensional axis, forming a charge transfer complex with the Co atoms, and the properties of the CoBHT (CO) lattice exhibits interesting magnetic properties, including spin-crossover behavior and long-range magnetic ordering. The TCNQ ligands provide a highly anisotropic electronic structure, which can result in highly directional exchange interactions between the Co atoms. The CO ligands can modulate the magnetic properties of the Co atoms by influencing their coordination environment and electronic structure. The CoBHT (CO) lattice has potential applications in magnetic data storage, spintronics, and molecular electronics. Overall, the CoBHT (CO) lattice is a promising molecular organic framework with unique magnetic properties and potential applications in various fields.

**Figure 3:**
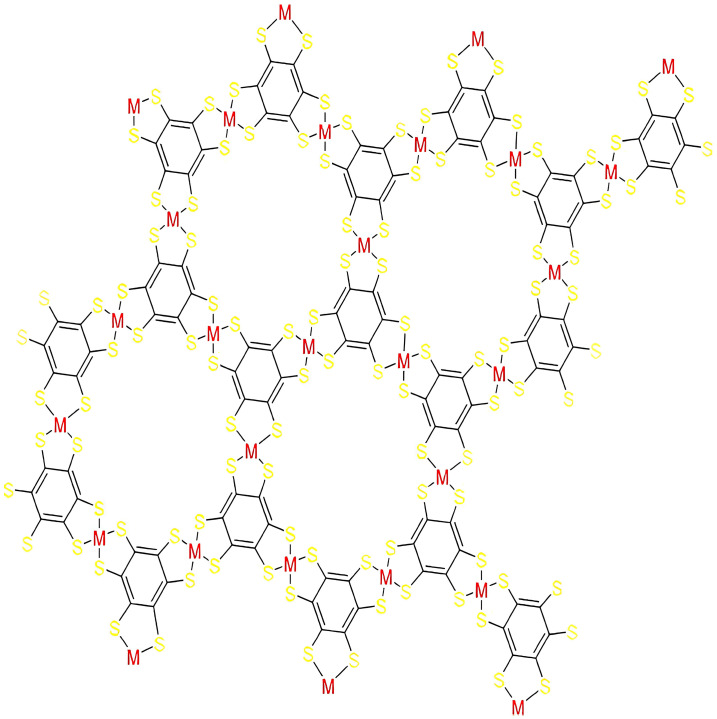
Supercell of 3 × 3 CoBHT (CO) lattice.

The C(g,h), a graph of CoBHT (CO) lattice, denotes the unit cell in the column and g denotes the unit cell in a row. The structure of the molecular graph of CoBHT (CO) lattice is shown in Figure 3, where the portion in a square shows the unit structure of CoBHT (CO) lattice. The T(g,h) has 27gh vertices and 36gh−2(g+h) edges. In Figure 3 two-dimensional 3×3CoBHT(CO) lattice structure is shown.

- **The 1st K-Banhatti entropy measure of ${\mathrm{CoBHT}}_{\left(g,h\right)}$**
  Let $CoBHT_{\left(g,h\right)}$ be a metal–organic framework. In view of Table 3 and Equation (1), we obtain
  (34) $$\begin{array}{ccc} B_{1}\left(CoBHT_{\left(g,h\right)},s\right)\; & = & 2\left(g+h\right)s^{4}+2\left(g+h\right)s^{2}+\left(12gh-2\left(g+h\right)\right)s^{5}\;\\ \; & + & \left(12gh\right)s^{6}+\left(12gh-2\left(g+h\right)\right)s^{6}. \end{array}$$
  After differentiating Equation (34) at s=1, we obtain
  (35) $$B_{1}\left(C\left(g,h\right)\right)=204gh-10\left(g+h\right).$$

**Table 3:** Atom-bonds partition of $CoBHT_{\left(g,h\right)}$.

Types of Atom Bonds	$E_{\left(1∼3\right)}$	$E_{\left(2∼2\right)}$	$E_{\left(2∼3\right)}$	$E_{\left(3∼3\right)}$	$E_{\left(2∼4\right)}$
Cardinality of Atom bonds	2(g+h)	2(g+h)	(12gh−2(g+h))	12gh	(12gh−2(g+h))
  The first *K*-Banhatti entropy measure of (C(g,h)) in view of Equations (10) and (35), Table 3:
  $$\begin{array}{ccc} ENT_{B_{1}}\left(C\left(g,h\right)\right) & = & \log\left(204gh-10(g+h)\right)\;\\ & - & \frac{1}{\left(204gh-10(g+h)\right)}\log\{2\left(g+h\right)4^{4}\;\\ & \times & 2\left(g+h\right)4^{4}\times \left(12gh-2\left(g+h\right)\right)5^{5}\;\\ & \times & \left(12gh\right)6^{6}\times \left(12gh-2\left(g+h\right)6^{6}\right)\}. \end{array}$$
- **The second K-Banhatti entropy measure of C(g,h)**
  The Equation (1) and Table 3, gives
  (36) $$\begin{array}{ccc} B_{2}\left(C\left(g,h\right),s\right) & = & 2\left(g+h\right)s^{3}+2\left(g+h\right)s^{4}+\left(12gh-2\left(g+h\right)\right)s^{6}\;\\ & + & 12ghs^{9}+\left(12gh-2\left(g+h\right)\right)s^{8}. \end{array}$$
  After differentiating Equation (36) at s=1, we have
  (37) $$B_{2}\left(C\left(g,h\right)\right)=276gh-14\left(g+h\right).$$
  The second *K*-Banhatti entropy measure of C(g,h) is obtained in view of Equations (11) and (37), Table 3:
  $$\begin{array}{ccc} ENT_{B_{2}}\left(C\left(g,h\right)\right) & = & \log\left(276gh-14(g+h)\right)\\ & - & \frac{1}{\left(276gh-14(g+h)\right)}\log\{(2\left(g+h\right)3^{3}\\ & \times & 2\left(g+h\right)4^{4}\times \left(12gh-2\left(g+h\right)\right)6^{6}\times \left(12gh\right)9^{9}\times \left(12gh-2\left(g+h\right)\right)8^{8}\}. \end{array}$$
- **The first K-hyper Banhatti entropy measure of C(g,h)**
  In view of Table 3 and Equation (3), we have
  (38) $$\begin{array}{ccc} HB_{1}\left(C\left(g,h\right),s\right) & = & 2\left(g+h\right)s^{16}+2\left(g+h\right)s^{16}+\left(12gh-2\left(g+h\right)\right)s^{25}\;\\ & + & \left(12gh\right)s^{36}+\left(12gh-2\left(g+h\right)\right)s^{36}. \end{array}$$
  After differentiating Equation (38) at s=1, we get
  (39) $$HB_{1}\left(C\left(g,h\right)\right)=1164gh-58\left(g+h\right).$$
  The first *K*-hyper Banhatti entropy measure of C(g,h) in view of Equations (12) and (39), Table 3:
  $$\begin{array}{ccc} ENT_{HB_{1}}\left(C\left(g,h\right)\right) & = & \log1164gh-58(g+h))-\frac{1}{1164gh-58(g+h)}\log\{2\left(g+h\right)4^{32}\\ & \times & 2\left(g+h\right)4^{32}\times \left(12gh-2\left(g+h\right)\right)5^{50}\times \left(12gh\right)6^{72}\times \left(12gh-2\left(g+h\right)\right)6^{72}. \end{array}$$
- **The second K-hyper Banhatti entropy measure of C(g,h)**
  In view of Table 3 and Equation (4), we get
  (40) $$\begin{array}{ccc} HB_{2}\left(C\left(g,h\right),s\right) & = & \sum_{\left(1,3\right)}s^{{\left(1\times 3\right)}^{2}}+\sum_{\left(2,2\right)}s^{{\left(2\times 2\right)}^{2}}+\sum_{\left(2,3\right)}s^{{\left(2\times 3\right)}^{2}}+\sum_{\left(3,3\right)}s^{{\left(3\times 3\right)}^{2}}+\sum_{\left(2,4\right)}s^{{\left(2\times 4\right)}^{2}}\;\\ & = & 2\left(g+h\right)s^{9}+2\left(g+h\right))s^{16}+\left(12gh--2\left(g+h\right)\right)s^{36}\;\\ & + & \left(12gh\right)s^{81}+\left(12gh-2\left(g+h\right)\right)s^{64}. \end{array}$$
  After differentiating Equation (40) at s=1, we have
  (41) $$HB_{2}\left(C\left(g,h\right)\right)=\frac{198}{7}+27\left(g+h\right).$$
  The second *K*-hyper Banhatti entropy measure of C(g,h) is obtained in view of Equation (41) Table 3 and Equation (13):
  $$\begin{array}{ccc} ENT_{HB_{2}}\left(C\left(g,h\right)\right) & = & \log\left(HB_{2}\right)-\frac{1}{HB_{2}}\log\{{\prod_{E_{\left(1,3\right)}}\left(w_{{\dot{g}}_{1}}\times w_{{\dot{g}}_{2}}\right)}^{2{\left(w_{{\dot{g}}_{1}}\times w_{{\dot{g}}_{2}}\right)}^{2}}\\ & \times & {\prod_{E_{\left(2,2\right)}}\left(w_{{\dot{g}}_{1}}\times w_{{\dot{g}}_{2}}\right)}^{2{\left(w_{{\dot{g}}_{1}}\times w_{{\dot{g}}_{2}}\right)}^{2}}\times {\prod_{E_{\left(2,3\right)}}\left(w_{{\dot{g}}_{1}}\times w_{{\dot{g}}_{2}}\right)}^{2{\left(w_{{\dot{g}}_{1}}\times w_{{\dot{g}}_{2}}\right)}^{2}}\\ & \times & {\prod_{E_{\left(3,3\right)}}\left(w_{{\dot{g}}_{1}}\times w_{{\dot{g}}_{2}}\right)}^{2{\left(w_{{\dot{g}}_{1}}\times w_{{\dot{g}}_{2}}\right)}^{2}}\times {\prod_{E_{\left(2,4\right)}}\left(w_{{\dot{g}}_{1}}\times w_{{\dot{g}}_{2}}\right)}^{2{\left(w_{{\dot{g}}_{1}}\times w_{{\dot{g}}_{2}}\right)}^{2}}. \end{array}$$
  This gives
  (42) $$\begin{array}{ccc} & = & \log\left(\frac{198}{7}+27\left(g+h\right)\right)-\frac{1}{\frac{198}{7}+27\left(g+h\right)}\log\{2\left(g+h\right)3^{18}\;\\ & \times & 2\left(g+h\right)4^{32}\times \left(12gh-2\left(g+h\right)\right)6^{72}\times \left(12gh\right)9^{162}\times \left(12gh-2\left(g+h\right)\right)8^{128}. \end{array}$$
- **The first redefined Zagreb entropy measure of C(g,h)**
  In view of Table 3 and Equation (5), we have
  (43) $$\begin{array}{ccc} ReZG_{1}\left(C\left(g,h\right),s\right) & = & \sum_{\left(1,3\right)}s^{\frac{1+3}{1\times 3}}+\sum_{\left(2,2\right)}s^{\frac{2+2}{2\times 2}}+\sum_{\left(2,3\right)}s^{\frac{2+3}{2\times 3}}+\sum_{\left(3,3\right)}s^{\frac{3+3}{3\times 3}}+\sum_{\left(2,4\right)}s^{\frac{2+4}{2\times 4}}\;\\ & = & 2\left(g+h\right)s^{\frac{4}{3}}+2\left(g+h\right)s^{\frac{4}{4}}+\left(12gh-2\left(g+h\right)\right)s^{\frac{5}{6}}\;\\ & + & \left(12gh\right)s^{\frac{6}{9}}+\left(12gh-2\left(g+h\right)\right)s^{\frac{6}{7}}. \end{array}$$
  After differentiating Equation (43) at s=1, we obtain the first redefined Zagreb index
  (44) $$ReZG_{1}\left(C\left(g,h\right)\right)=\frac{9}{7}\left(g+h\right)+\frac{198}{7}gh.$$
  The first redefined Zagreb entropy measure is obtained in view of Equation (44) Table 3 and Equation (14):
  $$\begin{array}{ccc} ENT_{ReZG_{1}}\left(C\left(g,h\right)\right) & = & \log\left(\frac{9}{7}\left(g+h\right)+\frac{198}{7}gh\right)\\ & - & \frac{1}{\left(\frac{9}{7}\left(g+h\right)+\frac{198}{7}gh\right)}\log\{2\left(g+h\right){\left(\frac{4}{3}\right)}^{\frac{4}{3}}\\ & \times & 2\left(g+h\right){\left(\frac{4}{4}\right)}^{\frac{4}{4}}\times \left(12gh-2\left(g+h\right)\right){\left(\frac{5}{6}\right)}^{\frac{5}{6}}\times 12gh{\left(\frac{6}{9}\right)}^{\frac{6}{9}}\\ & \times & \left(12gh-2\left(g+h\right)\right){\left(\frac{6}{8}\right)}^{\frac{6}{8}}\}. \end{array}$$
- **The second redefined Zagreb entropy measure of C(g,h)**
  In view of Table 3 and Equation (6), we obtain
  (45) $$\begin{array}{ccc} ReZG_{2}\left(C\left(g,h\right),s\right) & = & 2\left(g+h\right)s^{\frac{3}{4}}+2\left(g+h\right)s^{\frac{4}{4}}+\left(12gh-2\left(g+h\right)\right)s^{\frac{6}{5}}\;\\ & + & \left(12gh\right)s^{\frac{9}{6}}+\left(12gh-2\left(g+h\right)\right)s^{\frac{8}{6}}. \end{array}$$
  After differentiating Equation (45) at s=1, we obtain
  (46) $$ReZG_{2}\left(C\left(g,h\right)\right)=\frac{493}{30}\left(g+h\right)+\frac{112}{5}gh.$$
  The second redefined Zagreb entropy measure is obtained in view of Equations (15) and (46), Table 3:
  $$\begin{array}{ccc} ENT_{ReZG_{2}}\left(C\left(g,h\right)\right) & = & \log\left(\frac{493}{30}\left(g+h\right)+\frac{112}{5}gh\right)\\ & - & \frac{1}{\left(\frac{493}{30}\left(g+h\right)+\frac{112}{5}gh\right)}\log\{2\left(g+h\right){\left(\frac{3}{4}\right)}^{\frac{3}{4}}\times 2\left(g+h\right){\left(\frac{4}{4}\right)}^{\frac{4}{4}}\\ & \times & \left(12gh-2\left(g+h\right)\right){\left(\frac{6}{5}\right)}^{\frac{9}{6}}\times \left(12gh\right){\left(\frac{9}{6}\right)}^{\frac{9}{6}}\times \left(12gh-2\left(g+h\right)\right)\left(\frac{8}{6}\right)\}. \end{array}$$
- **The third redefined Zagreb entropy measure of C(g,h)**
  In view of Table 3 and Equation (7), we get
  $$\begin{array}{ccc} ReZG_{3}\left(C\left(g,h\right),s\right) & = & \sum_{\left(1,3\right)}s^{\left(1\times 3)(1+3\right)}+\sum_{\left(2,2\right)}s^{\left(2\times 2)(2+2\right)}+\sum_{\left(2,3\right)}s^{\left(2\times 3)(2+3\right)}\\ & + & \sum_{\left(3,3\right)}s^{\left(3\times 3)(3+3\right)}+\sum_{\left(2,4\right)}s^{\left(2\times 4)(2+4\right)}\\ & = & 2\left(g+h\right)s^{12}+2\left(g+h\right)s^{16}+\left(12gh-2\left(g+h\right)\right)s^{30}\\ & + & \left(12gh\right)s^{54}+\left(12gh-2\left(g+h\right)\right)s^{48}. \end{array}$$
  (47) $$\begin{array}{ccc} ReZG_{3}\left(C\left(g,h\right),s\right) & = & 2\left(g+h\right)s^{12}+2\left(g+h\right)s^{16}+\left(12gh-2\left(g+h\right)\right)s^{30}\\ & + & \left(12gh\right)s^{54}+\left(12gh-2\left(g+h\right)\right)s^{48}. \end{array}$$
  After differentiating Equation (47) at s=1, we obtain the third redefined Zagreb index
  (48) $$ReZG_{2}\left(C\left(g,h\right)\right)=-100\left(g+h\right)+1584gh.$$
  The third redefined Zagreb entropy measure is obtained in view of Equation (48) Table 3 and Equation (16):
  $$\begin{array}{ccc} ENT_{ReZG_{3}}\left(C\left(g,h\right),s\right) & = & \log\left(-100(g+h)+1584gh\right)-\frac{1}{\left(-100(g+h)+1584gh\right)}\log\{\left(24st+1\right)12^{12}\\ \; & \times & 6\left(s+t-1\right)30^{30}\times 2\left(28st-2s-2t+1\right)54^{54}\times 4\left(2st-s-t+1\right)84^{84}\}. \end{array}$$
- **Atom-bond sum connectivity entropy measure of C(g,h)**
  In view of Table 1 and Equation (8), the atom-bond sum connectivity polynomial is
  $$\begin{array}{ccc} ABS(C(g,h),s) & = & \sum_{\left(1,3\right)}s^{\sqrt{\frac{1+3-2}{1+3}}}+\sum_{\left(2,2\right)}s^{\sqrt{\frac{2+2-2}{2+2}}}+\sum_{\left(2,3\right)}s^{\sqrt{\frac{2+3-2}{2+3}}}+\sum_{\left(3,3\right)}s^{\sqrt{\frac{3+3-2}{3+3}}}\\ & + & \sum_{\left(2,4\right)}s^{\sqrt{\frac{2+4-2}{2+4}}}\;\\ & = & 2\left(g+h\right)s^{\frac{1}{\sqrt{2}}}+2\left(g+h\right)s^{\sqrt{\frac{3}{5}}}+\left(12gh-2\left(g+h\right)\right)s^{\sqrt{\frac{2}{3}}}\;\\ & + & 12ghs^{\sqrt{\frac{5}{7}}}+\left(12gh-2\left(g+h\right)\right)s^{\sqrt{\frac{2}{3}}}. \end{array}$$
  After differentiating Equation (49) at s=1, we have
  (49) $$\begin{array}{ccc} ABS(C(g,h)) & = & \sqrt{2}\left(g+h\right)+2\sqrt{\frac{3}{5}}\left(g+h\right)\\ & + & 2\sqrt{\frac{2}{3}}\left(12gh-2\left(g+h\right)\right)+12\sqrt{\frac{5}{7}}gh. \end{array}$$
  The third redefined Zagreb entropy measure is obtained in view of Equation (49), Table 3 and Equation (17):
  (50) $$\begin{array}{ccc} ENT_{ABS}\left(C\left(g,h\right)\right) & = & \log\left(ABS\right)-\frac{1}{ABS}\log\{2\left(g+h\right){\left(\frac{1}{\sqrt{2}}\right)}^{\frac{1}{\sqrt{2}}}\times 2\left(g+h\right){\left(\sqrt{\frac{3}{5}}\right)}^{\sqrt{\frac{3}{5}}}\;\\ \; & \times & \left(12gh-2\left(g+h\right)\right){\left(\sqrt{\frac{2}{3}}\right)}^{\sqrt{\frac{2}{3}}}\times 12gh{\left(\sqrt{\frac{5}{7}}\right)}^{\sqrt{\frac{5}{7}}}\times \left(12gh-2\left(g+h\right)\right){\left(\sqrt{\frac{2}{3}}\right)}^{\sqrt{\frac{2}{3}}}\}. \end{array}$$

### Comparison

**Figure 4:**
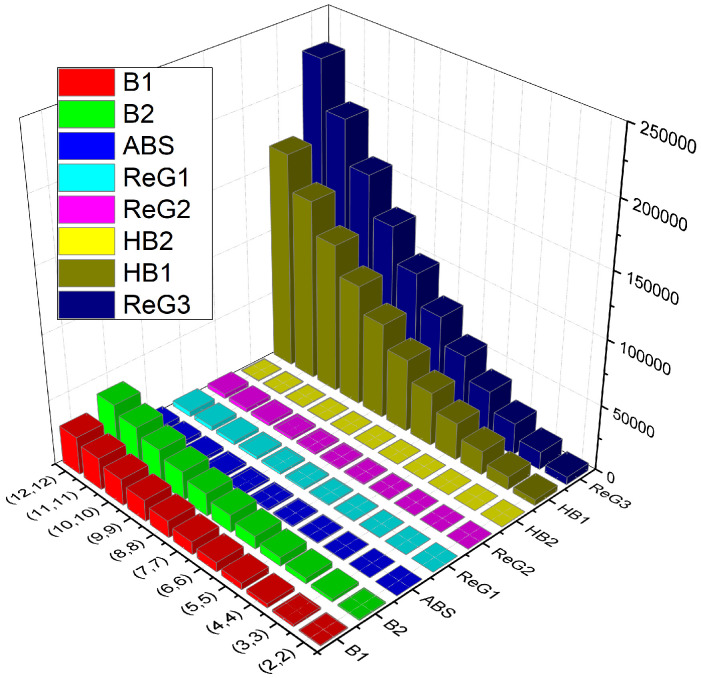
Graphical comparison of TIs of C(g,h).

In this section, we present a comparison (numerical in Table 4 and graphical in Figure 4) of various *K*-Banhatti and redefined Zagreb indices for C(g,h).

## 5. Conclusions

MOFs’ allure stems from their distinct qualities, which can be predicted and modified. MOF synthesis and analysis employ a diverse set of current scientific methodologies and procedures. Because of the amazing structural diversity observed in MOFs, these methods allow scientists to predict and regulate the properties of synthesised materials. The ability to tailor the structure of MOFs enables the development of materials with specialised properties for certain applications. The amazing optical attributes of metallic nanoparticles have piqued the curiosity of researchers and scientists of this era. In this study, the CoBHT (CO) lattice and the iron(III) tetra-p-tolyl porphyrin (FeTPyP), two significant metal–organic frameworks, have been investigated and using the atom-bond partitioning strategy, the precise formulas of numerous significant valency-based topological indices have been determined. The CoBHT (CO) lattice has potential applications in magnetic data storage, spintronics, and molecular electronics. Overall, the CoBHT (CO) lattice is a promising molecular organic framework with unique magnetic properties and potential applications in various fields. In this study, we also looked at the distance-based entropies related to a novel information function and evaluated the association between degree-based topological indices and degree-based entropies in light of Shannon’s entropy and Chen et al.’s entropy. This has been utilized to determine the complexity of molecules and molecular ensembles as well as their electrical structure, signal processing, physicochemical reactions, and complexity. The *K*-Banhatti entropy may be utilized in combination with thermodynamic entropy, chemical structure, energy, and mathematics to fill in gaps across various fields of study and build the foundation for new interdisciplinary research. This will open up new avenues for research in this field, as we plan to apply this concept to diverse metal organic frameworks in the future.

## Figures and Tables

**Table 1:** Atom-bond partition of FeTPyP-Co.

Types of Atom Bonds	$E_{\left(1∼3\right)}$	$E_{\left(2∼3\right)}$	$E_{\left(3∼3\right)}$	$E_{\left(3∼4\right)}$
Cardinality	24gh+1	6g+6h−6	56gh−4g−4h+2	8gh−4g−4h+4

**Table 2:** Numerical comparison of the computed indices of T(g,h).

(g,h)	$B_{1}$	$B_{2}$	${\mathrm{HB}}_{1}$	${\mathrm{HB}}_{2}$	${\mathrm{ReG}}_{1}$	${\mathrm{ReG}}_{2}$	${\mathrm{ReG}}_{3}$	ABS
(2,2)	1754	2790	22,074	20,259	294.67	436.88	14,070	270.90
(3,3)	4090	6414	510,66	48,411	664.67	1004.14	32,922	614.18
(4,4)	7402	11,526	92,202	88,371	1182.67	1802.83	59,742	1096.37
(5,5)	11,690	18,126	145,482	140,139	1848.67	2832.94	94,530	1717.47
(6,6)	16,954	26,214	210,906	203,715	2662.67	4094.48	137,286	2477.48
(7,7)	23,194	35,790	288,474	279,099	3624.67	5587.46	188,010	3376.41
(8,8)	30,410	46,854	378,186	366,291	4734.67	7311.86	246,702	4414.25
(9,9)	38,602	59,406	480,042	465,291	5992.67	9267.68	313,362	5590.99
(10,10)	47,770	73,446	594,042	576,099	7398.67	11,454.94	387,990	6906.65
(11,11)	57,914	88,974	720,186	698,715	8952.67	13,873.63	470,586	8361.22
(12,12)	69,034	105,990	858,474	833,139	10,654.67	16,523.74	561,150	9954.70

**Table 4:** Numerical comparison of the topological indices of C(g,h).

(g,h)	$B_{1}$	$B_{2}$	${\mathrm{HB}}_{1}$	${\mathrm{HB}}_{2}$	${\mathrm{ReG}}_{1}$	${\mathrm{ReG}}_{2}$	${\mathrm{ReG}}_{3}$	ABS
(2,2)	776	1048	4424	136.28	118.28	155.33	5936	117.74
(3,3)	1776	2400	10,128	190.28	262.28	300.2	13,656	265.82
(4,4)	3184	4304	18,160	244.28	462.86	489.86	24,544	473.38
(5,5)	5000	6760	28,520	298.28	720	724.33	38,600	740.42
(6,6)	7224	9768	41,208	352.28	1033.71	1003.6	55,824	1066.93
(7,7)	9856	13,328	56,224	406.28	1404	1327.66	76,216	1452.91
(8,8)	12,896	17,440	73,568	460.28	1830.86	1696.53	99,776	1898.37
(9,9)	16,344	22,104	93,240	514.28	2314.28	2110.2	126,504	2403.31
(10,10)	20,200	27,320	115,240	568.28	2854.28	2568.66	156,400	2967.72
(11,11)	24,464	33,088	139,568	622.28	3450.86	3071.93	189,464	3591.61
(12,12)	29,136	39,408	166,224	676.28	4104	3620	225,696	4274.97

## Data Availability

All data generated or analyzed during this study are included in this article.
